# Fast Detection of Snakes and Emotional Faces in the Macaque Amygdala

**DOI:** 10.3389/fnbeh.2022.839123

**Published:** 2022-03-21

**Authors:** Ha Trong Dinh, Yang Meng, Jumpei Matsumoto, Tsuyoshi Setogawa, Hiroshi Nishimaru, Hisao Nishijo

**Affiliations:** ^1^System Emotional Science, Faculty of Medicine, University of Toyama, Toyama, Japan; ^2^Department of Physiology, Vietnam Military Medical University, Hanoi, Vietnam; ^3^Research Center for Idling Brain Science (RCIBS), University of Toyama, Toyama, Japan

**Keywords:** macaque, amygdala, single-unit activity, snakes, emotional faces

## Abstract

Primate vision is reported to detect snakes and emotional faces faster than many other tested stimuli. Because the amygdala has been implicated in avoidance and emotional behaviors to biologically relevant stimuli and has neural connections with subcortical nuclei involved with vision, amygdalar neurons would be sensitive to snakes and emotional faces. In this study, neuronal activity in the amygdala was recorded from Japanese macaques (*Macaca fuscata*) during discrimination of eight categories of visual stimuli including snakes, monkey faces, human faces, carnivores, raptors, non-predators, monkey hands, and simple figures. Of 527 amygdalar neurons, 95 responded to one or more stimuli. Response characteristics of the amygdalar neurons indicated that they were more sensitive to the snakes and emotional faces than other stimuli. Response magnitudes and latencies of amygdalar neurons to snakes and monkey faces were stronger and faster than those to the other categories of stimuli, respectively. Furthermore, response magnitudes to the low pass-filtered snake images were larger than those to scrambled snake images. Finally, analyses of population activity of amygdalar neurons suggest that snakes and emotional faces were represented separately from the other stimuli during the 50–100 ms period from stimulus onset, and neutral faces during the 100–150 ms period. These response characteristics indicate that the amygdala processes fast and coarse visual information from emotional faces and snakes (but not other predators of primates) among the eight categories of the visual stimuli, and suggest that, like anthropoid primate visual systems, the amygdala has been shaped over evolutionary time to detect appearance of potentially threatening stimuli including both emotional faces and snakes, the first of the modern predators of primates.

## Introduction

Rapid defensive responses are often required to avoid predators. Neural systems for such behaviors have been argued to be controlled by an evolved subcortical “fear module” that in mammals includes the superior colliculus (SC) and pulvinar, two nuclei involved with vision, and the amygdala (Öhman and Mineka, 2001; Isbell, 2006, 2009). Both the SC and pulvinar receive visual inputs from the retinal ganglion cells, while the pulvinar receives visual inputs from the superficial and deep layers of the SC (Soares et al., 2017; Isa et al., 2021). These retinal ganglion cells projecting to the SC mainly convey magnocellular (M) and kinocellular (K) channels of visual information with low spatial resolution (Waleszczyk et al., 2004; Grünert et al., 2021). The pulvinar further projects to at least lateral part of the amygdala in macaques (Elorette et al., 2018). This SC-pulvinar-amygdalar pathway appears to unconsciously detect appearance/movement of potentially threatening stimuli including phylogenetically salient stimuli in shorter latencies than the cortical visual system (Tamietto and de Gelder, 2010; Soares et al., 2017; McFadyen et al., 2020). Anthropoid primates (including humans) are highly dependent on vision for detecting danger. It has been argued that, as the first modern predator of primates, snakes have long exerted strong evolutionary pressure on primate visual systems to increase sensitivity to visual cues provided by snakes (Isbell, 2006, 2009). Growing evidence consistently supports this. For example, naïve monkeys and humans detect snakes more quickly than other objects under a variety of conditions (Nelson et al., 2003; Kawai and Koda, 2016; Bertels et al., 2020).

As highly social mammals, anthropoid primates must also respond quickly to conspecifics, especially when threatened. The subcortical “fear module” has been implicated in the ability of people with cortical lesions showing affective blindsight to unconsciously discriminate emotional faces (Morris et al., 2001; de Gelder et al., 2005; Pegna et al., 2005; Tamietto et al., 2012). A neurophysiological study reported that monkey pulvinar neurons in macaque monkeys responded more quickly and more strongly to both of these phylogenetically salient stimuli than to neutral objects (monkey hands and geometric shapes), as expected of nuclei in the “fear module” (Le et al., 2013). It is likely that the pulvinar’s biased responses for images of snakes and emotional faces arose via the superior colliculus as the pulvinar receives visual inputs from the superior colliculus (Le et al., 2013). In rodents, the amygdala, the other core structure of the “fear module,” plays a crucial role in mediating defensive responses to unconditioned stimuli such as predator-relevant stimuli (Martinez et al., 2011; Root et al., 2014; Isosaka et al., 2015). However, the responsiveness of amygdalar neurons to phylogenetically salient stimuli in primates, especially snakes, is currently unknown.

In this study, we attempted to close this gap in knowledge by comparing neuronal responses to snakes and emotional faces as salient stimuli against other presumably less salient visual stimuli in the amygdala of the Japanese macaque (*Macaca fuscata*) as a model for anthropoid primates. To analyze amygdalar neuronal sensitivity to innate biological significance of visual stimuli, especially snakes, amygdalar neurons were tested with eight categories of the visual stimuli [snakes, monkey faces (emotional and neutral faces), human faces (emotional and neutral faces), monkey hands, non-predators, raptors, carnivores, and simple figures]. The main predators of primates are carnivores, raptors, and snakes, of which snakes are the first of the modern predators of primates (Isbell, 1994), suggesting that snakes might shape the visual system of primates for a longer time than the other predators. Facial stimuli including emotional faces were also included as visual stimuli to test amygdalar neurons, since these stimuli were reported to be biologically salient (Öhman et al., 2001; Hershler and Hochstein, 2005; Hahn et al., 2006; Langton et al., 2008). Non-predators and simple figures were used as control stimuli that are supposed to have less innate biological significance. However, stimuli with physical salience such as onset/offset of intense light, to which the subcortical visual pathway are also sensitive (Itti and Koch, 2001; Yoshida et al., 2012), were not used in the present study. Amygdalar neurons were also tested with low- and high-pass filtered snake images as well as scrambled snake images. The subcortical pathway including the amygdala, which processes fast and coarse information, is reported to be more responsive to low-pass filtered stimuli (Vuilleumier et al., 2003; Le et al., 2013; Gomes et al., 2018). Here, we report that macaque amygdalar neurons responded faster and stronger to snakes than to other potential predators for primates, and that they responded faster and stronger to emotional faces compared to neutral faces. The results also indicated that amygdalar neurons were more sensitive to low spatial frequency components of snake images compared to high spatial frequency components of those stimuli, and scrambling of snake images decreased the responses. These results suggest that, like the pulvinar, the anthropoid primate amygdala has evolved to receive fast and coarse visual information about evolutionarily salient stimuli that have the potential to affect primate survival.

## Materials and Methods

### Subjects

Two adult male Japanese macaques (Monkey1 and Monkey2), weighing 7.3 and 8.6 kg (6 years old at the time of training and recording), were used in this experiment. Both monkeys were not used in the previous experiments including Le et al. (2013) and Dinh et al. (2018). They were born in a breeding facility in the Primate Research Institute in Kyoto University, Japan, and reared in a group cage (5–6 monkeys per cage: Monkey1), where one side of the cage was in contact with the external environment and monkeys could freely see outside, or in a colony in an outer enclosed area (Monkey2). They were transferred to University of Toyama at the age of three, and individually housed with free access to monkey chow and water. They were also given vegetables and fruits. Environmental enrichment (toys) was provided daily. They experienced no stressful procedures for the whole duration. The monkeys were deprived of water in their home cages during training and recording sessions and received supplemental water and vegetables after each day’s session at the age of six. The monkeys were treated according to the Guidelines for the Care and Use of Laboratory Animals of the University of Toyama as well as the United States Public Health Service Policy on Human Care and Use of Laboratory Animals, the National Institutes of Health Guide for the Care and Use of Laboratory Animals. According to Japanese law, the Committee for Animal Experiments and Ethics at the University of Toyama reviewed and approved the experimental protocol and procedures in this study.

### Visual Stimuli

**FIGURE 1:**
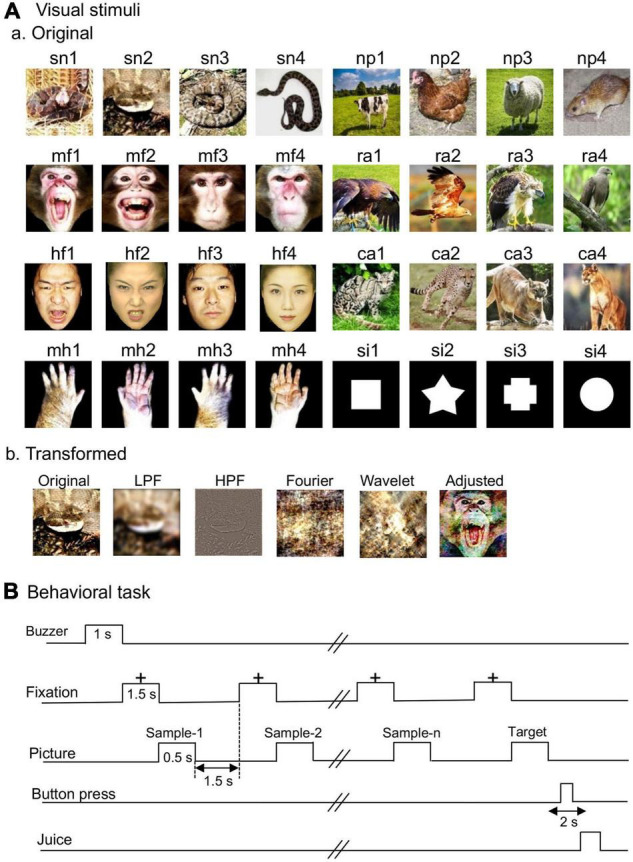
Visual stimuli **(A)** and delayed non-matching-to-sample (DNMS) task **(B)**. (Aa) Non-transformed original stimuli. Thirty-two photos of eight categories of the visual stimuli including snakes, monkey faces (emotional and neutral faces), human faces (emotional and neutral faces), monkey hands, non-predators, raptors, carnivores, and simple figures. (Ab) Transformed stimuli. LPF, low-pass filtered images; HPF, high-pass filtered images; Fourier, Fourier-scrambled images; Wavelet, wavelet-scrambled images; Adjusted, adjusted visual images. **(B)** Schematic illustration of the DNMS task sequence. The photos are used on the courtesy of Mr. D. Hillman (sn1), Mr. I. Hoshino (sn2-4), Mr. Rich Lindie (ra3), Mr. Prasanna De Alwis (ra4), EarthWatch Institute (ca2), Microsoft Windows98 (ca4); “Brahminy Kite juvenile” (ra2) by Challiyan is licensed under CC BY-SA 3.0; “cow” (np1) and “chicken” (np2) are freely available pictures from Pixabay; “sheep” (np3) was purchased from Pixabay; “The black rat” (np4) by CSIRO is licensed under CC BY-SA 3.0. Human facial pictures (hf2, hf4) were purchased from ATR-Promotions (rights holder of the pictures). Copyright: ATR-Promotions, republished with permission.

The same visual stimuli that were used in a previous study (Dinh et al., 2018) were used in this study except that a cat in the previous study was replaced with a cow in the present study. Figure 1Aa shows eight categories of the visual stimuli used in the present study: snakes, monkey faces (emotional and neutral faces), human faces (emotional and neutral faces), monkey hands, non-predators, raptors, carnivores, and simple figures.

The color stimuli were 8-bits digitized RGB color-scale images (227 × 227 pixels). The luminance of the color stimuli was very similar (6.005–6.445 cd/m^2^). The luminance of the white areas inside the simple figures was 36.5 cd/m^2^. Michelson contrast was not significantly different among the eight categories of the visual stimuli (Dinh et al., 2018). These stimuli were presented at the center of a display (resolution: 640 × 480 pixels) with a black background of 0.7 cd/m^2^. The size of the stimulus area was 5–7 × 5–7°.

### Transformed Visual Stimuli

The transformed visual stimuli were introduced to analyze the characteristics of the stimuli that amygdalar neurons responded to Figure 1Ab. According to previous studies (Vuilleumier et al., 2003; Rotshtein et al., 2007), a low-pass filter (LPF: 6 cycles/image) and a high-pass filter (HPF: 20 cycles/image) were used to modify spatial frequencies of the stimuli. The original visual stimuli were processed in the same way as a previous study (Le et al., 2013) to obtain the low- and high-pass filtered images.

Following a previous study (Honey et al., 2008), the visual images were scrambled using two different methods (Fourier and Wavelet scrambling). In Fourier scrambling, the positions (i.e., phases) of all spatial frequency components were randomized while the Fourier 2-D amplitude spectrum across orientations and spatial frequencies were maintained. In wavelet scrambling, the orientations of local spatial frequency components were randomized while the local wavelet coefficient values were maintained. These transformed visual stimuli maintained almost completely the global low-level properties of the original images (i.e., global contrast, luminance, spatial frequency amplitude spectrum, color) (Honey et al., 2008). These images were transformed using MatLab.

Some visual stimuli were transformed (adjusted visual stimuli) using the SHINE toolbox on MatLab (Willenbockel et al., 2010; Railo et al., 2016) so that low-level properties (the color histogram and the amplitude spectra of each color channel of RGB) of the visual stimuli were equalized with means of the low-level properties of the snake stimuli. However, the equalization of the properties cannot be perfect because of limitations in the method (Willenbockel et al., 2010). Therefore, to determine if the equalization removed differences of low-level properties among the stimulus categories, the following low-level properties of the adjusted stimuli were statistically compared (Le et al., 2016). Michelson contrast was compared among the five categories by one-way ANOVA (the categories: the original snakes, adjusted raptors, adjusted human faces, adjusted monkey faces, and adjusted carnivores). Michelson contrast of each category of the adjusted stimuli was also directly compared with the original snake stimuli by unpaired *t*-test. A color histogram consisting of four equally spaced bins was calculated for each color channel. Each bin of the histograms was statistically compared with one-way ANOVA and unpaired *t*-test in the same way as described above. Power spectrum averaged overall orientation (Simoncelli and Olshausen, 2001) was calculated for each color channel. The total power of low and high spatial frequency was calculated as the total power in the power spectrum from 1 to 8 cycles/image and from 9 to 113 cycles/image, respectively (Le et al., 2016). Each total power of high or low spatial frequency was statistically compared with one-way ANOVA and unpaired *t*-test in the same way as described above. The results of statistical comparisons indicated that none of the statistically significant difference was found in the tests (see Supplementary Tables 1, 2 for the details).

### Behavioral Task

The procedures are detailed in our previous studies (Le et al., 2013; Dinh et al., 2018). Briefly, a 19-inch computer display was placed 68 cm away from a monkey chair for a behavioral task. The monkey’s eye position of the left eye was monitored by an eye-monitoring system using a CCD camera (Matsuda, 1996). The timing of visual stimulus onset and juice delivery was controlled by a visual stimulus control unit (ViSaGe MKII Visual Stimulus Generator, Cambridge Research Systems).

The monkeys were required to perform a delayed non-matching-to-sample task (DNMS) to ensure that the monkeys looked at visual stimuli not associated with reward in the same condition (i.e., in the central visual field without saccadic eye movements). In the DNMS, the monkeys compared and discriminated the visual stimuli presented in sequence (Figure 1B). In the task, a fixation cross appeared in the center of the monitor after a buzzer tone. After fixating to the cross for 1.5 s, a sample stimulus appeared for 500 ms (sample phase). The same stimulus appeared again for 500 ms between 1 and 4 times after each 1.5-s interval of fixation. Finally, a new target stimulus appeared (target phase), and the monkeys pressed a button within 2 s to obtain a juice reward (0.8 mL). When the monkeys did not correctly press the button within the 2 s in the target phase, the trials terminated and a buzzer tone (620-Hz) was presented. The trials were repeated with intertrial intervals of 15–25 s.

Thus, the monkeys compared a pair of stimuli (i.e., sample vs. target stimuli) in each trial. The stimuli were randomly selected within the same stimulus category.

### Training and Surgery

The monkeys were trained to perform the DNMS task for 3 h/day, 5 days/week so that performance of the monkeys was asymptotic. All visual stimuli were presented a similar number of times to the monkeys only during training and recording sessions. After 3 months of training, the monkeys reached a 90% correct-response rate. Then, a head-restraining device (U-frame made of reinforced plastic) to receive fake ear-bars was implanted on the skull under anesthesia induced by medetomidine (0.5 mg/kg, i.m.)—ketamine (5 mg/kg, i.m.) combination (Nishijo et al., 1988a,b; Tazumi et al., 2010; Dinh et al., 2018). The U-frame was anchored with dental acrylic to titanium screws twisted into the skull. A small reference pin was also implanted, and its position was calibrated according to the coordinates of the stereotaxic atlas of the *M. fuscata* brain (Kusama and Mabuchi, 1970). Antibiotics (Ceftriaxone sodium, 20 mg/kg) was administered for 1 week after the surgery. After a recovery period of 2 weeks after the surgery, the monkeys were retrained with their heads painlessly fixed to the stereotaxic apparatus to reach performance criterion (>90%).

### Neurophysiological Recording Procedures

The procedures were essentially the same as in earlier studies (Le et al., 2013; Dinh et al., 2018). Briefly, a glass-insulated tungsten microelectrode (0.5–1.5 MΩ at 1 kHz), the tip location of which was calibrated from the reference pin, was stereotaxically inserted into the amygdala with a micromanipulator (Narishige, Tokyo, Japan) according to the stereotaxic atlas of the *M. fuscata* brain (Kusama and Mabuchi, 1970). Neuronal activity with an S/N ratio greater than 3:1 was recorded. All data including neuronal activity, trigger signals of task events, and the coordinates of eye positions were stored in a computer via a data processor (MAP; Plexon, Dallas) system. After recording of responses to the 32 original visual stimuli, the neurons were further tested with the transformed images if the single-unit activity was still observed.

Trials, when eye locations deviated larger than 1.0 degrees from the fixation cross during the fixation and sample periods, were excluded from the analysis. The stored neuronal activities were sorted into single units using the Offline Sorter program for cluster analysis (Plexon). After isolation of clusters, an autocorrelogram for each isolated unit was constructed to observe refractory periods, and units with refractory periods greater than 1.2 ms were selected. Finally, superimposed waveforms of the isolated units throughout the recording sessions were assessed to check variability during recording. These isolated units were further analyzed by the NeuroExplorer program (Nex Technologies, MA, United States) (see below).

### Statistical Procedures for Analyzing Amygdalar Neuron Responses

The data were analyzed as in two previous studies (Le et al., 2013; Dinh et al., 2018). The data only during the sample phase, but not the target phase, were analyzed. Significant (excitatory or inhibitory) responses to each stimulus were defined by comparison of neuronal activity during the following two periods [Wilcoxon signed-rank (WSR) test, *p* < 0.05]: 100 ms before (pre) and 500 ms after (post) the stimulus onset. Visually responsive neurons were defined as those that responded to one or more visual stimuli (WSR test, *p* < 0.05).

In each responsive neuron, the mean response magnitudes to each stimulus category were computed. Each responsive neuron was categorized by the best category that elicited the largest responses. For example, “snake-best” neurons were those that showed larger mean response magnitude to all snake images than those to the remaining stimulus categories. In each responsive neuron, the mean response magnitudes to each stimulus were computed. Then, the grand mean response magnitudes of the four stimuli in each category were compared among the eight stimulus categories by one-way ANOVA (*p* < 0.05) with Tukey *post hoc* tests (*p* < 0.05). Furthermore, mean response magnitudes of all responsive neurons (*n* = 95) were compared among the eight stimulus categories by repeated measures one-way ANOVA (*p* < 0.05) with Bonferroni *post hoc* tests (*p* < 0.05). In the above analyses of response magnitudes and best-stimulus category, absolute values were used for neurons with inhibitory responses (*n* = 4).

Response latencies to the eight stimulus categories were also analyzed. First, for each neuron, each peri-event histogram for each stimulus category was constructed using the entire set of data for all trials for each stimulus category. Then, neuronal response latency was estimated as the duration between the stimulus onset and the moment at which neuronal activity exceeded the mean ± 2 SD of neuronal activity during the 100-ms pre-period before the stimulus onset. The mean latencies to the eight stimulus categories of all neurons, in which latencies to all eight categories could be estimated using the above criterion (*n* = 75), were compared by repeated measures one-way ANOVA (*p* < 0.05) with Bonferroni *post hoc* tests (*p* < 0.05).

To analyze the effects of facial expression (neutral vs. emotional) and species (monkeys vs. humans) on amygdalar neuronal activity, response magnitudes and latencies to the individual monkey and human faces were similarly estimated. Then, mean response magnitudes and latencies (*n* = 95 for response magnitudes; *n* = 75 for response latencies) to the monkey and human faces were analyzed by repeated measures two-way ANOVA (*p* < 0.05) with expression and species as factors.

Finally, response magnitudes to individual stimuli of the snakes, monkey faces, monkey hands, and simple figures were estimated to analyze relationships between the amygdala (present data) and pulvinar (Le et al., 2013) by a linear regression analysis. All data were expressed as mean ± SEM. In ANOVA statistical analyses, sphericity was assessed, and the degrees of freedom were corrected by Greenhouse-Geisser method where appropriate. All statistical analyses were conducted using SPSS (v. 28, IBM) and GraphPad Prism v. 9.2.0 (GraphPad Software, San Diego, CA).

### Multidimensional Scaling Analysis

The data were analyzed as in two earlier studies (Le et al., 2013; Dinh et al., 2018). To investigate stimulus representation in the amygdala, data matrices of response magnitudes during specific periods after stimulus onset were analyzed by multidimensional scaling (MDS). MDS creates a geometric representation of stimuli based on the relationship (dissimilarity in the present study) between stimuli to quantitatively analyze similarity of groups of given items (see Hout et al., 2013 for more details). Items are placed in MDS space so that items with similar response patterns are located proximally. Each dimension of the MDS space represents a different underlying factor to estimate similarity.

The data in four 50-ms epochs comprising the initial 200-ms post period were analyzed using MDS. In each 50-ms epoch, the data matrix of response magnitudes in a 95 × 32 array derived from the 95 visually responsive neurons was generated. The response magnitude in each epoch was computed as the mean firing rate in each epoch subtracted by the mean firing rate during the 100-ms pre-period before the stimulus onset. In MDS, the MDS program (SPSS, v. 28) initially computed dissimilarity (Euclidean distances) between all pairs of two visual stimuli based on the response magnitudes of the 95 amygdalar neurons. Then, the program placed the 32 visual stimuli in the stimulus space so that the distribution of the stimuli in the MDS space represented the original relationships between the stimuli (i.e., Euclidean distances in the present study) (Shepard, 1962). Finally, the separation of clusters of the visual stimuli in the stimulus space was assessed by discriminant analyses.

### Localization of the Recording Sites

The animals were treated as in a previous study (Dinh et al., 2018). Before recording, MRI scans of the monkey head with a tungsten marker (diameter, 800 μm) inserted near the recording region were taken under anesthesia. After the last recording, tungsten markers (diameter, 500 μm) were implanted near the recording sites under anesthesia. Then, the monkeys were anesthetized (sodium pentobarbital, 100 mg/kg, i.m.) and perfused initially with 0.9% saline, and then with 10% buffered formalin. After perfusion, the brains including the amygdala were cut into 100-μm coronal sections, which were stained with Cresyl violet. The locations of the marker tips were determined microscopically, and the location of each recording site was determined by comparing the stereotaxic coordinates of recording sites and those of maker positions.

In the present study, anatomical classification and nomenclature of the amygdalar subnuclei followed the stereotaxic atlas of the *M. fuscata* brain (Kusama and Mabuchi, 1970). According to the previous studies (Nishijo et al., 1988a,b), the amygdala was divided into four areas; CM, corticomedial group of the amygdala (CM), lateral nucleus (AL), basolateral nucleus (Abl), and basomedial nucleus (Abm).

## Results

### Responses to Eight Categories of the Visual Stimuli

**FIGURE 2:**
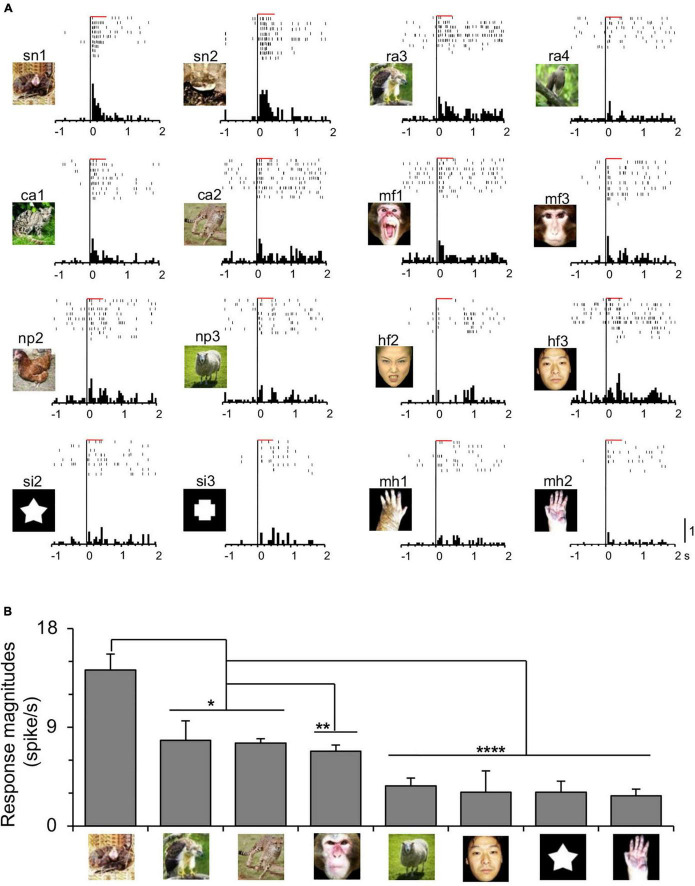
Neuronal responses of a snake-responsive amygdalar neuron. **(A)** Neuronal responses to each indicated stimulus are shown by raster displays and their summed histograms. Horizontal red bars above the raster displays, stimulus presentation periods (500 ms); bin width of histograms, 50 ms; calibration at the right bottom of the figure, number of spikes per trial in each bin. **(B)** Comparison of response magnitudes of the neuron shown in **(A)** to the eight categories of the visual stimuli. ****, **, *, *p* < 0.0001, 0.01, 0.05, respectively (Tukey test after one-way ANOVA). Copyright: ATR-Promotions, republished with permission.

Of 1,253 amygdala neurons recorded from Monkey1 (M1) (*n* = 1,151) and Monkey2 (M2) (*n* = 102), 527 were tested with the eight categories of the non-transformed visual stimuli (M1, *n* = 486; M2, *n* = 41). Of these 527, 95 neurons responded to one or more visual stimuli (visually responsive neurons) (M1, *n* = 88; M2, *n* = 7). Figure 2 shows an amygdalar neuron that responded selectively to snakes. The neuron responded strongly to snakes and less to other stimuli (Figure 2A). The mean response magnitudes (grand mean responses of mean responses to each stimulus in each stimulus category) of the neuron significantly differed among the stimulus categories [one-way ANOVA: *F*_(7, 24)_ = 10.7, *p* < 0.0001] (Figure 2B), and the mean response magnitudes to snakes were significantly greater than those to the other stimulus categories including raptors and carnivores (Tukey test, *p* < 0.05), monkey faces (Tukey test, *p* = 0.0043), and non-predators, human faces, simple figures, and monkey hands (Tukey test, *p* < 0.0001). To compare the response trends to the visual stimuli between the two monkeys (Monkey1 and Monkey2), mean response magnitudes were compared among the eight categories in each monkey (Supplementary Figure 1). In Monkey1, response magnitudes to snakes, monkey faces, and human faces were significantly stronger to the other stimulus categories (Supplementary Figure 1A). In Monkey2, a comparable response trend was observed: response magnitudes to snakes and monkey faces were significantly stronger to the other stimulus categories except for human faces (Supplementary Figure 1B). Furthermore, mean responses to each visual stimulus were significantly and positively correlated (Supplementary Figure 2). These results indicate that the response trends were similar between the two monkeys. Therefore, in the following analyses, the combined data of the two monkeys were analyzed.

**FIGURE 3:**
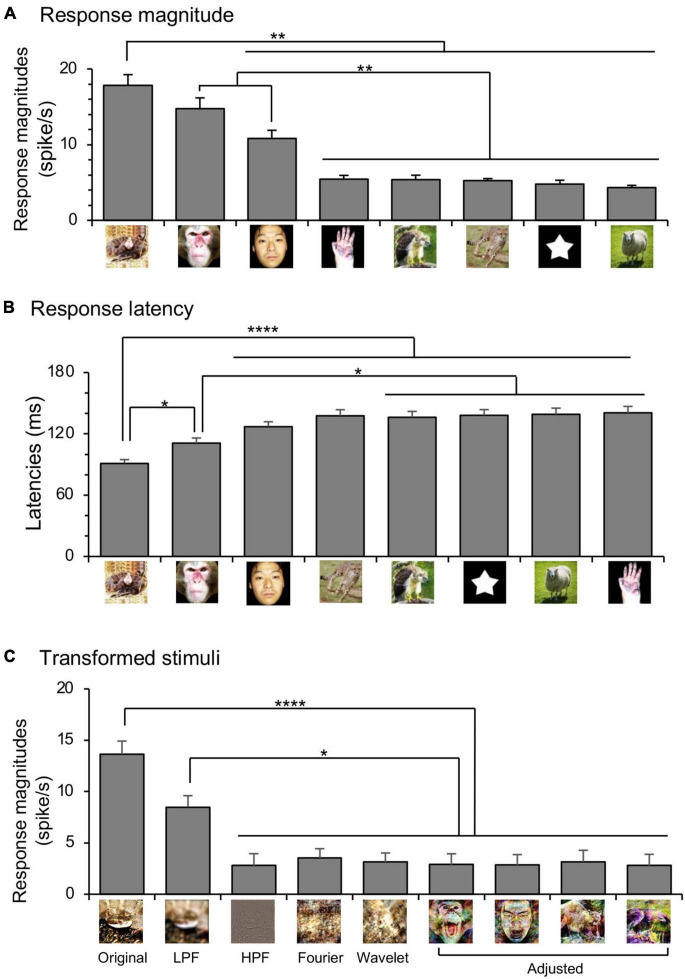
Response characteristics of the amygdala neurons to the eight categories of the visual stimuli **(A,B)** and the transformed stimuli **(C)**. **(A)** Comparison of the mean response magnitudes to the eight stimulus categories. **, *p* < 0.01, respectively (Bonferroni test after repeated measures one-way ANOVA). **(B)** Comparison of the mean response latencies to the eight stimulus categories. ****, *, *p* < 0.0001, 0.05, respectively (Bonferroni test after repeated measures one-way ANOVA). **(C)** Comparison of averaged response magnitudes of 26 amygdalar neurons to the original and transformed images. Original, original snake images; LPF, low pass-filtered snake images; HPF, high pass-filtered snake images; Fourier, Fourier-scrambled snake images; Wavelet, Wavelet-scrambled snake images; Adjusted, adjusted images. ****, *, *p* < 0.0001, 0.05, respectively (Bonferroni test).

The amygdalar neurons were categorized by “best-category” that elicited the largest responses (Le et al., 2013). Of the 95 visually responsive neurons, snake-best neurons were most frequent (*n* = 28), followed by monkey face-best neurons (*n* = 26; 27.4%), and human face-best neurons (*n* = 21; 22.1%). Mean response magnitudes of the 95 visually responsive neurons significantly differed among the eight stimulus categories [repeated measures one-way ANOVA: *F*_(3.831, 360.1)_ = 36.05, *p* < 0.0001] (Figure 3A). The mean response magnitudes were significantly stronger to snakes than to the remaining stimuli except for the monkey faces (Bonferroni test, *p* < 0.01). Furthermore, the mean response magnitudes were significantly stronger to the monkey and human faces than to the monkey hands, raptors, carnivores, simple figures, and non-predators (Bonferroni test, *p* < 0.01).

Response latencies of the amygdalar neurons ranged from 40 to 415 ms (*n* = 75). Mean response latencies also significantly differed among the eight categories [repeated measures one-way ANOVA: *F*_(5.999, 443.9)_ = 10.80, *p* < 0.0001] (Figure 3B). The mean response latency was significantly shorter for the snakes than for the monkey faces (Bonferroni test, *p* < 0.05), and the human faces, carnivores, raptors, simple figures, non-predators, and monkey hands (Bonferroni test, *p* < 0.0001). Furthermore, the mean response latencies were significantly shorter for the monkey faces than for the raptors, simple figures, non-predators, and monkey hands (Bonferroni test, *p* < 0.05).

The 26 snake-best neurons were further tested with not only the original snake images but also the transformed images including the low-pass filtered snake images, high-pass filtered snake images, Fourier-scrambled snake images, Wavelet-scrambled snake images, and adjusted monkey face images, adjusted human face images, adjusted carnivore images, and adjusted raptor images (Figure 3C). The mean response magnitudes significantly differed among these nine stimulus groups [repeated measures one-way ANOVA: *F*_(5.835, 145.9)_ = 12.92, *p* < 0.0001], and the mean response magnitude to the original snakes were significantly greater than those to other stimuli except for the low pass-filtered snake images (Bonferroni test, *p* < 0.0001). Furthermore, the mean response magnitude to the low pass-filtered snake images was significantly larger than those to the high pass-filtered snake images, scrambled snake images (Fourier, Wavelet), and adjusted images of the monkey faces, human faces, carnivores, and raptors (Bonferroni test, *p* < 0.05).

### Responses to Emotional Faces

**FIGURE 4:**
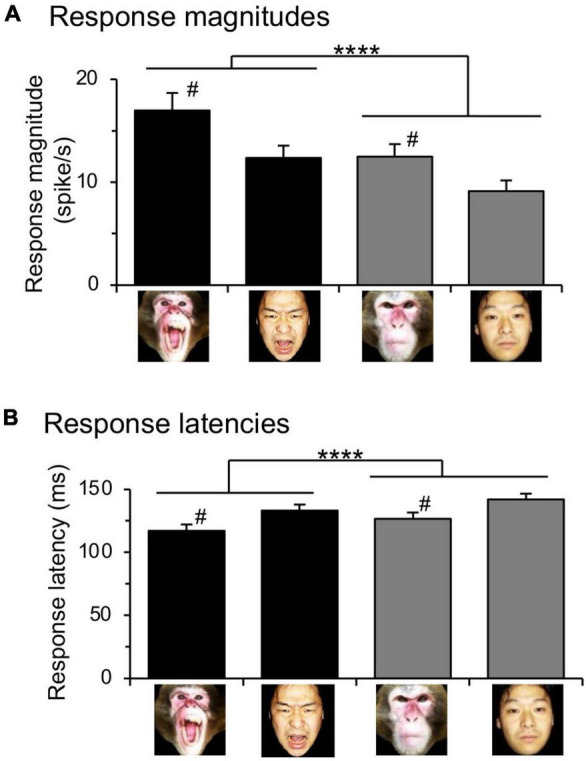
Effects of expression and species of monkey and human faces on amygdalar neuronal responses. **(A)** Comparison of the mean response magnitudes. ****p < 0.0001 (significant main effect of expression: emotional vs. neutral); ^#^*p* < 0.05 (significant main effect of species: monkey vs. human). **(B)** comparison of the mean response latencies. ****p < 0.0001 (significant main effect of expression: emotional vs. neutral); ^#^*p* < 0.05 (significant main effect of species: monkey vs. human).

Previous studies reported that emotional faces, but not neutral faces, were preferentially processed in the subcortical visual pathway (Vuilleumier et al., 2003; Le et al., 2013; Diano et al., 2017). To investigate this possibility in the monkey amygdala, mean response magnitudes to the monkey and human faces were analyzed with repeated measures two-way ANOVAs with species (monkey vs. human) and expression (emotional vs. neutral) as factors (Figure 4A). The statistical results indicated significant main effects of species [*F*_(1.0, 94.0)_ = 5.810, *p* = 0.0179] and expression [*F*_(1.0, 94.0)_ = 88.86, *p* < 0.0001], but no significant interaction between these two factors [*F*_(1.0, 94.0)_ = 2.039, *p* = 0.1567]. Mean response latencies to the monkey and human faces were similarly analyzed (Figure 4B). The statistical results of repeated measures two-way ANOVAs indicated significant main effects of expression [*F*_(1.0, 74.0)_ = 89.34, *p* < 0.0001] and species [*F*_(1.0, 74.0)_ = 5.792, *p* = 0.0186], but no significant interaction between the two factors [*F*_(1.0, 74.0)_ = 0.1274, *p* = 0.721].

### Relationships to the Macaque Pulvinar Neurons

**FIGURE 5:**
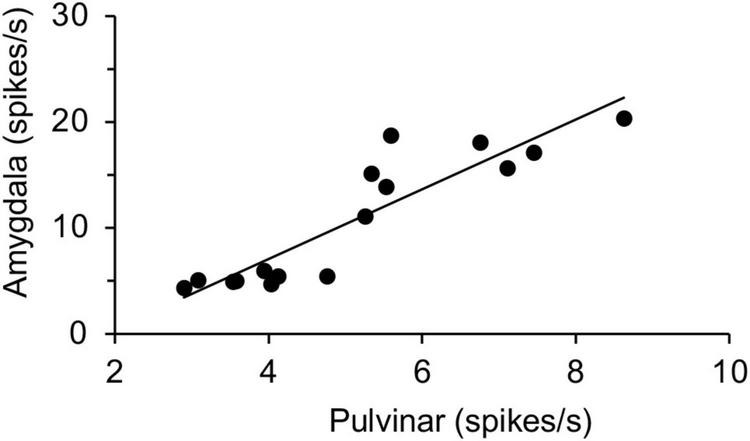
Linear correlation of response magnitudes between the pulvinar and amygdala. Response magnitudes to the same 16 stimuli in the amygdala and those in the pulvinar (Le et al., 2013) are plotted. There was a significant positive correlation between the amygdala and pulvinar in response magnitudes (*p* < 0.0001).

Thus, the amygdalar neurons responded stronger and faster to snakes and emotional faces, which was very similar to the previous study on the macaque pulvinar neurons (Le et al., 2013). Previous anatomical studies reported that the amygdala receives visual information of threating stimuli from the SC-pulvinar in tree shrews and macaques (Day-Brown et al., 2010; Elorette et al., 2018). If the subcortical visual pathway is involved in processing of innate biological significance of visual stimuli, we hypothesized that activity of amygdalar neurons would be correlated to activity of the pulvinar neurons even in different monkeys. To investigate possible relationships between the amygdala and pulvinar, the correlation between neuronal responses in the pulvinar (Le et al., 2013) and those in the amygdala (present study) was analyzed (Figure 5), since this and previous studies used the same visual stimuli (a total of 16 stimuli: snakes, monkey faces, monkey hands, and simple figures) tested in the same DNMS task. The results indicated that response magnitudes were significantly and positively correlated between the amygdala and pulvinar [simple regression analysis: *r*^2^ = 0.820; *F*_(1, 14)_ = 63.80, *p* < 0.0001].

### Stimulus Representation in the Amygdala

**FIGURE 6:**
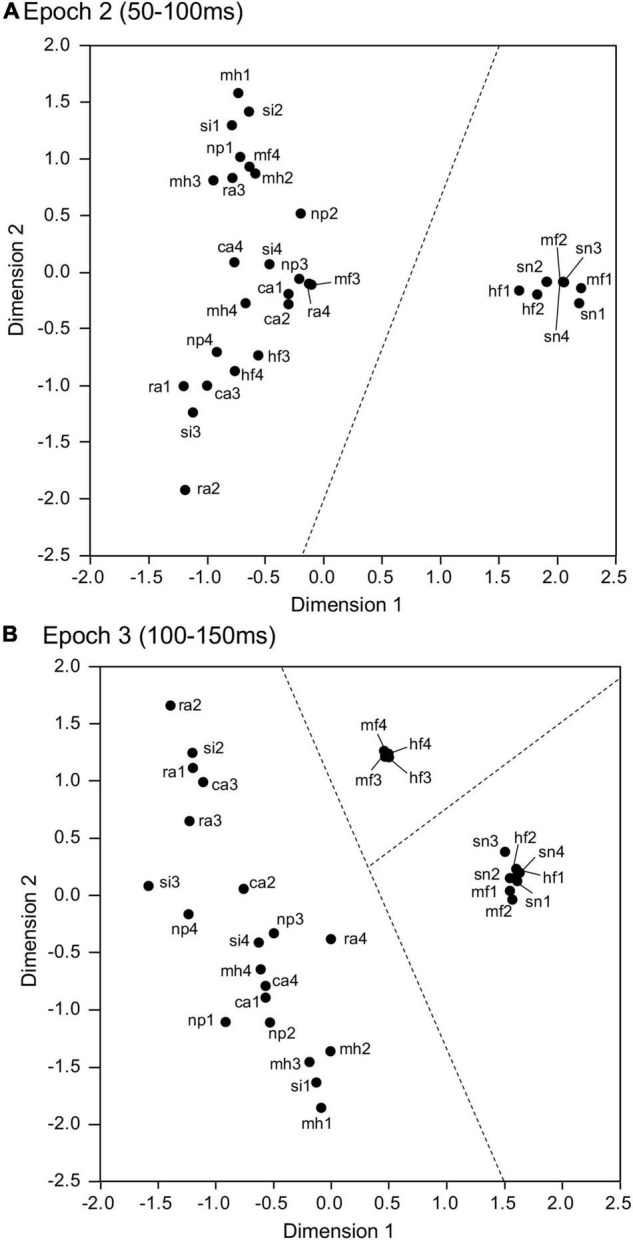
Representation of the 32 visual stimuli in a 2D space resulting from MDS using responses of the 95 amygdalar neurons to these stimuli. **(A)** Representation in epoch 2. The snakes (sn1-4) and emotional faces (mf1-2 and hf1-2) were separated from the remaining stimuli. **(B)** Representation in epoch 3. Three groups were separated: group 1 (snakes and emotional faces: sn1-4, mf1-2, and hf1-2), group 2 (neutral faces: mf3-4 and hf3-4), and group 3 containing the remaining stimuli.

The four data matrices in epochs 1 (0–50 ms), 2 (50–100 ms), 3 (100–150 ms), and 4 (150–200 ms) after stimulus onset, each consisting of response magnitudes to the 32 visual stimuli in the individual 95 visually responsive amygdalar neurons, were separately analyzed using MDS. In the resultant 2D spaces, *r*^2^-values of epochs 1, 2, 3, and 4 were 0.481, 0.784, 0.618, and 0.730, respectively. In epoch 2 (Figure 6A), two groups were observed: a cluster containing the snakes (sn1-4) and emotional faces (mf1-2, hf1-2), and another containing the remaining stimuli (non-snake and non-emotional face images). These two clusters (snakes and emotional faces vs. non-snakes and non-emotional faces) were significantly separated (discriminant analysis: Wilks’ lambda = 0.338, *p* < 0.0001). In epoch 3 (Figure 6B), three groups were observed; a cluster containing the snakes (sn1-4) and emotional faces (mf1-2, hf1-2); a cluster containing the neutral faces (mf3-4, hf3-4), and a cluster containing all the other stimuli. These three clusters were also significantly separated (discriminant analysis: Wilks’ lambda = 0.155, *p* < 0.0001). These MDS findings indicated that the activity patterns of the population amygdalar neurons discriminated snakes, emotional faces, and neutral faces. However, in epoch 1 (Supplementary Figure 3) and epoch 4 (Supplementary Figure 4), no significant stimulus cluster was observed.

### Locations of the Amygdalar Neurons

**FIGURE 7:**
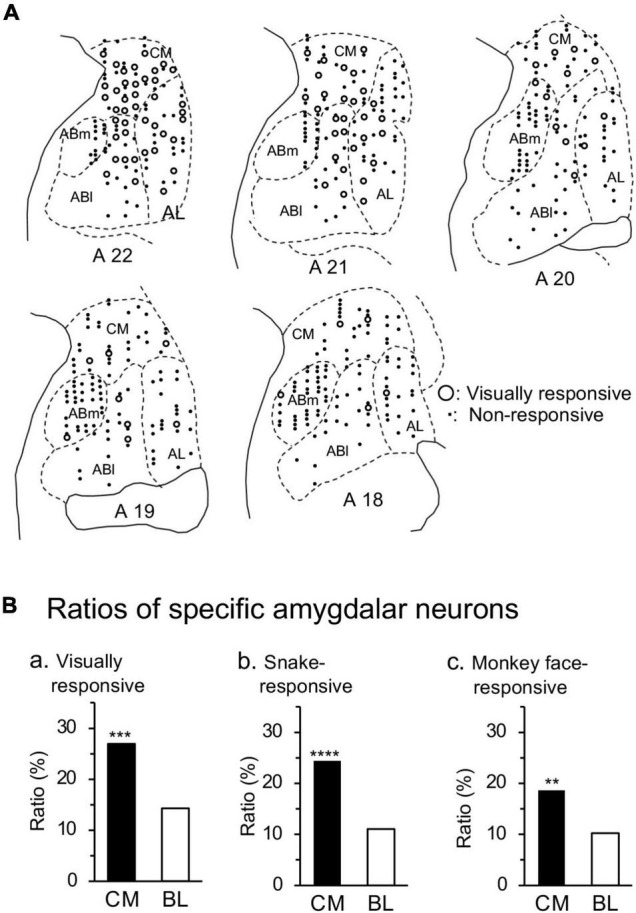
Recording sites in the amygdala. **(A)** Recording sites are plotted on coronal sections at the different anteroposterior axis (A-P levels). Numbers below coronal sections, distance (mm) anteriorly from the interaural line; open circles, responsive neurons (*n* = 95); dots, non-responsive neurons. The amygdala was divided into four areas: CM, corticomedial group; AL, lateral nucleus; Abl, basolateral nucleus; Abm, basomedial nucleus. **(B)** Ratios of responsive neurons from all the recorded neurons in the corticomedial and basolateral groups of the amygdala. The ratios of the visually responsive (a), snake-responsive (b), and monkey face-responsive (c) neurons were significantly greater in the corticomedial group of the amygdala than in the basolateral group of the amygdala. ****, ***, **, *p* < 0.0001, 0.001, 0.01 (χ^2^-test).

Figure 7A shows the recording sites of responsive neurons in the amygdala. The amygdala was divided into four areas: corticomedial (CM) group of the amygdala, and basolateral (BL) group of the amygdala including the lateral (AL), basolateral (ABl), and basomedial (ABm) nuclei. The ratios of the responsive neurons differed significantly between the corticomedial and basolateral groups (Figure 7B). The ratios of the visually responsive neurons from all the recorded neurons were significantly greater in the corticomedial group than the basolateral group (χ^2^-test: χ^2^ = 11.87, *p* = 0.0006). Furthermore, ratios of the neurons that responded significantly to snakes from all the recorded neurons were significantly larger in the corticomedial group than the basolateral group (χ^2^-test: χ^2^ = 15.26, *p* < 0.0001). In addition, ratios of the neurons that responded significantly to monkey faces from all the recorded neurons were significantly larger in the corticomedial group than the basolateral group (χ^2^-test: χ^2^ = 6.895, *p* = 0.0086).

## Discussion

In the present study, we found that amygdalar neurons responded selectively to snake and face stimuli: (i) mean response magnitudes for snakes, monkey faces, and human faces were greater than those for other stimuli; (ii) mean response latencies for snakes and monkey faces were shorter than for other stimuli; and (iii) activity patterns of the 95 amygdalar responsive neurons discriminated snakes and emotional faces within a 100-ms latency. These responses to snakes were dependent at least on low-frequency components of the images, while high-pass filtering and scrambling of the snake images decreased neuronal responses. The present results provide neurophysiological evidence that amygdalar neurons receive fast and coarse visual information of fear-relevant stimuli (snakes and emotional faces), which may be effective in eliciting rapid behavioral responses in response to those stimuli in nature (see below).

### Responsiveness of Amygdalar Neurons

Consistent with studies of the “fear module” where snakes and emotional faces are particularly salient stimuli in humans (Morris et al., 2001; Almeida et al., 2015), the present study indicated greater responsiveness of amygdalar neurons to snakes and emotional faces than to other predators (carnivores and raptors) and neutral stimuli. Fourier- and Wavelet-scrambling of the snake images decreased responses to the snakes. In addition, responses to the adjusted images of the monkey and human faces, carnivores, and raptors, in which low-level properties of the images were equalized with means of the low-level properties of the snake images, were smaller than those to the original snake images. These findings suggest that the high responsiveness to snakes is less dependent on global low-level properties of the original snake stimuli, but more dependent on the form of the snakes.

High spatial, but not low spatial, filtering of snake images decreased response magnitudes to the snakes, suggesting that preferential responsiveness to the snake images was dependent on its low spatial frequency component in the present study. Consistently, high spatial, but not low spatial, filtering of snake images decreased response magnitudes to the snakes in the monkey pulvinar that projects to the amygdala (Le et al., 2013). Furthermore, low spatial frequency images of emotional faces elicited fast synaptic responses in the human amygdala in early latencies (less than 100 ms) before cortical responses (Méndez-Bértolo et al., 2016). Human fMRI studies are also consistent with the present results: low spatial component of emotional faces elicited greater responses in the amygdala than the low spatial component of neutral faces in a patient with V1 lesions and intact humans (Vuilleumier et al., 2003; Burra et al., 2019). These findings suggest that the amygdala receives crude features of dangerous visual signals faster than the visual cortex and consequently is involved in coarse and fast processing of such signals, which might be crucial for self-defense and survival.

### Detection of Snakes and Emotional Faces in the Amygdala

Previous behavioral studies consistently reported that amygdalar lesions decreased aversive behavioral responses to snakes in monkeys and human patients (Meunier et al., 1999; Kalin et al., 2001; Izquierdo et al., 2005; Mason et al., 2006; Machado et al., 2009; Feinstein et al., 2011), and disturbed recognition of emotional faces in human patients (Adolphs et al., 1994; Calder et al., 1996). Our MDS results indicated that snakes and emotional faces were separated from the other stimuli in the second epoch (50–100 ms after stimulus onset), suggesting a pivotal role of the amygdala in fast detection of emotional stimuli. This early time window strongly suggests that the amygdala directly receives visual inputs from the subcortical pathway bypassing the cortical routes. In contrast, neutral faces were separated in the third epoch (100–150 ms after stimulus onset). This suggests that cortical inputs such as those from temporal face areas may also contribute to amygdalar neuronal responses to facial stimuli (Pitcher et al., 2017).

A recent monkey behavioral study reported that bilateral selective lesions of the central nucleus of the amygdala also disturbed aversive behavioral responses to snakes (Kalin et al., 2004). Consistent with this finding, most human fMRI studies reported that emotional stimuli activated the amygdala, especially its dorsal or dorsomedial part (Breiter et al., 1996; de Gelder et al., 2005; Schaefer et al., 2014; Hakamata et al., 2016), which roughly corresponds to the corticomedial group of the amygdala that includes the central, medial, and cortical nuclei of the amygdala. The amygdala is functionally divided into two regions with reciprocal connections (Aggleton, 1985; Bielski et al., 2021): the dorsomedial part that corresponds to the corticomedial group of the amygdala (output nuclei for behavioral responses), and the ventrolateral part that corresponds to the basolateral group of the amygdala (affective evaluation of sensory stimuli). Consistent with the above fMRI studies, the present study indicated that the ratios of the responsive neurons were significantly larger in the corticomedial group than the basolateral group of the amygdala. This suggests that the corticomedial group of the amygdala is more responsive to various types of aversive stimuli, and consequently, it may be involved with stereotyped first response, subconscious, subcortically controlled behaviors (e.g., avoidance, freezing, etc.), whereas the basolateral group may be more involved with later, cortically controlled evaluation and decision-making (McGarry and Carter, 2017; Ishikawa et al., 2020).

In the present study, all visual stimuli were similarly presented to the monkeys only during training and recording sessions. Therefore, the differences in responses among the eight categories are likely to be attributed to their innate biological significance, consistent with previous studies reporting that behavioral responses to snakes and faces are innate in monkeys (for snakes: Nelson et al., 2003; Kawai and Koda, 2016; Bertels et al., 2020; for faces: Sackett, 1966; Sugita, 2008). However, previous studies also reported that behavioral responses to these stimuli may be altered by prior experience: macaque monkeys developed a fear of snakes by observational learning (Cook and Mineka, 1990), and stressful early life experience of parents of subjects might alter coping styles of the subjects in response to snakes in marmosets (Clara et al., 2008). Although they did not go through such stressful procedures before or during the present study, they interacted directly with their conspecifics in the breeding facility and encountered humans during training and recording. Furthermore, we could not exclude the possibility that the monkeys might have encountered real snakes at the breeding facility. However, the amygdalar responses to the stimuli in the present study were significantly correlated to the pulvinar responses in the previous study in which the monkeys were unlikely to have encountered real snakes (Le et al., 2013). These findings suggest that amygdalar responses to snakes were attributed to their innate biological significance.

However, there are several limitations in the present study. First, this study was designed to analyze effects of innate biological significance (biological saliency) of the static visual stimuli, but not stimulus saliency of low-grade visual properties (physical saliency, i.e., luminance, motion, and color of the stimulus). It has been reported that these physical properties of visual stimuli are also critical as stimulus saliency in patients with V1 lesions (Itti and Koch, 2001; Yoshida et al., 2012), suggesting that the subcortical visual pathway is sensitive to such stimuli. Therefore, stimuli such as onset/offset of light with different intensities could affect amygdalar neuronal activity, and the present results do not deny such possibility. We speculate that the subcortical visual pathway may be sensitive to both biological and physical saliency of visual stimuli. Second, we tested amygdalar neurons only with a limited number of static visual images. The results of scrambling the snake images only indicate that stimulus coherency is important for neuronal sensitivity to the snake images. Thus, specific features of the snake images that activate amygdalar neurons were not analyzed in detail. In future studies, it is interesting to compare neuronal activity between sinusoidal line moving and a snake slither pattern, and to test snake-responsive neurons with a branch of similar thickness and color pattern and Gaussian light onset/offset, etc.

### Role of the Subcortical Pathway in Primates

The above discussion suggests that amygdalar neuronal responsiveness is similar to that in the pulvinar and medial prefrontal cortex in monkeys (Maior et al., 2010; Le et al., 2013; Dinh et al., 2018). This specific pattern of responsiveness to snakes and emotional faces might be attributed to natural selection favoring rapid detection of snakes as well as emotional faces in primates via the “fear module,” which includes the superior colliculus, pulvinar, and amygdala (Isbell, 2006, 2009; Öhman et al., 2012).

Snakes were the first modern predators of primates (and other mammals), having evolved before carnivorans and raptors, and, because many snakes are sit-and-wait predators, they can often be avoided by initially freezing or jumping away, suggesting that snakes may have been a main driver behind subcortical visual evolution (Isbell, 2006, 2009). In the present study, response magnitudes were positively correlated between the pulvinar and amygdala, consistent with a human fMRI study which reported that activity in the amygdala and pulvinar was positively correlated (Morris et al., 2001; Troiani et al., 2014), suggesting that this subcortical pathway is functional in intact monkeys. A monkey anatomical study also indicated that amygdalar neurons receive visual information from the superior colliculus through the medial part of the pulvinar, where snake-sensitive neurons were most common (Le et al., 2013), and which may contribute to rapid processing of emotional stimuli (Elorette et al., 2018). Furthermore, bilateral lesions of the superior colliculus abolished aversive behavioral responses to snakes in monkeys (Maior et al., 2011).

Almost all anthropoid primates live in groups and are highly social. Flexible facial expressions can help individuals determine the intentions of others (Burrows, 2008; Dobson, 2009; Dobson and Sherwood, 2011), which can range from highly affiliative to highly agonistic, the latter sometimes resulting in death (Martínez-Íñigo et al., 2021). Thus, responding rapidly to avoid a threatening conspecific may reduce injury and increase the chances of survival. As with snakes, emotional faces may have provided a reliable cue for avoiding danger and selective pressure for visual evolution (Soares et al., 2017).

Pulvinar lesions also disturbed unconscious processing of emotional stimuli in human patients (Bertini et al., 2018). In addition, fMRI studies reported that fiber density connecting the superior colliculus, pulvinar, and amygdala, detected using tractography, was positively correlated with functional connectivity between the pulvinar and amygdala, and positively correlated with recognition of emotional faces and attentional bias to threatening stimuli (Koller et al., 2019; McFadyen et al., 2019). These findings suggest that the amygdala’s role in behavioral responses to snakes and emotional faces in primates is dependent on visual inputs to the amygdala through the visual pathway of the subcortical “fear module.” The present results provide complementary neurophysiological evidence of the same visual dependency.

## Data Availability Statement

The original contributions presented in the study are included in the article/Supplementary Material, further inquiries can be directed to the corresponding author/s.

## Ethics Statement

The animal study was reviewed and approved by the Committee for Animal Experiments and Ethics at the University of Toyama. Written informed consent was obtained from the individual for the publication of any potentially identifiable images or data included in this article.

## Author Contributions

HsN and HrN conceived the study and designed the experiment. HD and YM performed the experiment. HD, YM, JM, and HsN analyzed data and wrote the manuscript. HsN, HrN, TS, and JM revised the manuscript. All authors discussed the results and commented on the manuscript, and read and approved the final manuscript.

## Conflict of Interest

The authors declare that the research was conducted in the absence of any commercial or financial relationships that could be construed as a potential conflict of interest.

## Publisher’s Note

All claims expressed in this article are solely those of the authors and do not necessarily represent those of their affiliated organizations, or those of the publisher, the editors and the reviewers. Any product that may be evaluated in this article, or claim that may be made by its manufacturer, is not guaranteed or endorsed by the publisher.

## References

[B1] Adolphs R. Tranel D. Damasio H. Damasio A. (1994). Impaired recognition of emotion in facial expressions following bilateral damage to the human amygdala. *Nature* 372 669–672. 10.1038/372669a0 7990957

[B2] Aggleton J. P. (1985). A description of intra-amygdaloid connections in old world monkeys. *Exp. Brain Res.* 57 390–399. 10.1007/BF00236545 3972039

[B3] Almeida I. Soares S. C. Castelo-Branco M. (2015). The Distinct Role of the Amygdala, Superior Colliculus and Pulvinar in Processing of Central and Peripheral Snakes. *PLoS One* 10:e0129949. 10.1371/journal.pone.0129949 26075614 PMC4467980

[B4] Bertels J. Bourguignon M. de Heering A. Chetail F. De Tiège X. Cleeremans A. (2020). Snakes elicit specific neural responses in the human infant brain. *Sci. Rep.* 10:7443. 10.1038/s41598-020-63619-y 32366886 PMC7198620

[B5] Bertini C. Pietrelli M. Braghittoni D. Làdavas E. (2018). Pulvinar Lesions Disrupt Fear-Related Implicit Visual Processing in Hemianopic Patients. *Front. Psychol.* 9:2329. 10.3389/fpsyg.2018.02329 30524351 PMC6261973

[B6] Bielski K. Adamus S. Kolada E. Rączaszek-Leonardi J. Szatkowska I. (2021). Parcellation of the human amygdala using recurrence quantification analysis. *Neuroimage* 227:117644. 10.1016/j.neuroimage.2020.117644 33338610

[B7] Breiter H. C. Etcoff N. L. Whalen P. J. Kennedy W. A. Rauch S. L. Buckner R. L. (1996). Response and habituation of the human amygdala during visual processing of facial expression. *Neuron* 17 875–887. 10.1016/s0896-6273(00)80219-6 8938120

[B8] Burra N. Hervais-Adelman A. Celeghin A. de Gelder B. Pegna A. J. (2019). Affective blindsight relies on low spatial frequencies. *Neuropsychologia* 128 44–49. 10.1016/j.neuropsychologia.2017.10.009 28993236

[B9] Burrows A. M. (2008). The facial expression musculature in primates and its evolutionary significance. *Bioessays* 30 212–225. 10.1002/bies.20719 18293360

[B10] Calder A. J. Young A. W. Rowland D. Perrett D. I. Hodges J. R. Etcoff N. L. (1996). Facial emotion recognition after bilateral amygdala damage: differentially severe impairment of fear. *Cogn. Neuropsychol.* 13 699–745.

[B11] Clara E. Tommasi L. Rogers L. J. (2008). Social mobbing calls in common marmosets (Callithrix jacchus): effects of experience and associated cortisol levels. *Anim. Cogn.* 11 349–358. 10.1007/s10071-007-0125-0 18066607

[B12] Cook M. Mineka S. (1990). Selective associations in the observational conditioning of fear in rhesus monkeys. *J. Exp. Psychol. Anim. Behav. Process* 16 372–389. 2230660

[B13] Day-Brown J. D. Wei H. Chomsung R. D. Petry H. M. Bickford M. E. (2010). Pulvinar projections to the striatum and amygdala in the tree shrew. *Front. Neuroanat.* 4:143. 10.3389/fnana.2010.00143 21120139 PMC2991220

[B14] de Gelder B. Morris J. S. Dolan R. J. (2005). Unconscious fear influences emotional awareness of faces and voices. *Proc. Natl. Acad. Sci. U. S. A.* 102 18682–18687. 10.1073/pnas.0509179102 16352717 PMC1317960

[B15] Diano M. Celeghin A. Bagnis A. Tamietto M. (2017). Amygdala Response to Emotional Stimuli without Awareness: facts and Interpretations. *Front. Psychol.* 7:2029. 10.3389/fpsyg.2016.02029 28119645 PMC5222876

[B16] Dinh H. T. Nishimaru H. Matsumoto J. Takamura Y. Le Q. V. Hori E. (2018). Superior neuronal detection of snakes and conspecific faces in the macaque medial prefrontal cortex. *Cereb. Cortex* 28 2131–2145. 10.1093/cercor/bhx118 28498964

[B17] Dobson S. D. (2009). Socioecological correlates of facial mobility in nonhuman primates. *Am. J. Phys. Anthropol.* 139 413–420. 10.1002/ajpa.21007 19235791

[B18] Dobson S. D. Sherwood C. C. (2011). Correlated evolution of brain regions involved in producing and processing facial expressions in anthropoid primates. *Biol. Lett.* 7 86–88. 10.1098/rsbl.2010.0427 20591852 PMC3030864

[B19] Elorette C. Forcelli P. A. Saunders R. C. Malkova L. (2018). Colocalization of tectal inputs with amygdala-projecting neurons in the macaque pulvinar. *Front. Neural Circuits* 12:91. 10.3389/fncir.2018.00091 30405362 PMC6207581

[B20] Feinstein J. S. Adolphs R. Damasio A. Tranel D. (2011). The human amygdala and the induction and experience of fear. *Curr. Biol.* 21 34–38. 10.1016/j.cub.2010.11.042 21167712 PMC3030206

[B21] Gomes N. Soares S. C. Silva S. Silva C. F. (2018). Mind the snake: fear detection relies on low spatial frequencies. *Emotion* 18 886–895. 10.1037/emo0000391 29265840

[B22] Grünert U. Lee S. C. S. Kwan W. C. Mundinano I. C. Bourne J. A. Martin P. R. (2021). Retinal ganglion cells projecting to superior colliculus and pulvinar in marmoset. *Brain Struct. Funct.* 226 2745–2762. 10.1007/s00429-021-02295-8 34021395

[B23] Hahn S. Carlson C. Singer S. Gronlund S. D. (2006). Aging and visual search: automatic and controlled attentional bias to threat faces. *Acta Psychol.* 123 312–336. 10.1016/j.actpsy.2006.01.008 16524554

[B24] Hakamata Y. Sato E. Komi S. Moriguchi Y. Izawa S. Murayama N. (2016). The functional activity and effective connectivity of pulvinar are modulated by individual differences in threat-related attentional bias. *Sci. Rep.* 6:34777. 10.1038/srep34777 27703252 PMC5050502

[B25] Hershler O. Hochstein S. (2005). At first sight: a high-level pop out effect for faces. *Vision Res.* 45 1707–1724. 10.1016/j.visres.2004.12.021 15792845

[B26] Honey C. Kirchner H. VanRullen R. (2008). Faces in the cloud: fourier power spectrum biases ultrarapid face detection. *J. Vis.* 8 1–13. 10.1167/8.12.9 18831622

[B27] Hout M. C. Papesh M. H. Goldinger S. D. (2013). Multidimensional scaling. *Wiley Interdiscip. Rev. Cogn. Sci.* 4 93–103. 10.1002/wcs.1203 23359318 PMC3555222

[B28] Isa T. Marquez-Legorreta E. Grillner S. Scott E. K. (2021). The tectum/superior colliculus as the vertebrate solution for spatial sensory integration and action. *Curr. Biol.* 31 R741–R762. 10.1016/j.cub.2021.04.001 34102128 PMC8190998

[B29] Isbell L. A. (1994). Predation on primates: ecological patterns and evolutionary consequences. *Evol. Anthropol.* 3 61–71. 10.1002/evan.1360030207

[B30] Isbell L. A. (2006). Snakes as agents of evolutionary change in primate brains. *J. Hum. Evol.* 51 1–35. 16545427 10.1016/j.jhevol.2005.12.012

[B31] Isbell L. A. (2009). *Fruit, the Tree, and the Serpent: Why We See So Well.* Cambridge: Harvard University Press.

[B32] Ishikawa J. Sakurai Y. Ishikawa A. Mitsushima D. (2020). Contribution of the prefrontal cortex and basolateral amygdala to behavioral decision-making under reward/punishment conflict. *Psychopharmacology* 237 639–654. 10.1007/s00213-019-05398-7 31912190

[B33] Isosaka T. Matsuo T. Yamaguchi T. Funabiki K. Nakanishi S. Kobayakawa R. (2015). Htr2a-Expressing Cells in the Central Amygdala Control the Hierarchy between Innate and Learned Fear. *Cell* 163 1153–1164. 10.1016/j.cell.2015.10.047 26590419

[B34] Itti L. Koch C. (2001). Computational modelling of visual attention. *Nat. Rev. Neurosci.* 2 194–203. 10.1038/35058500 11256080

[B35] Izquierdo A. Suda R. K. Murray E. A. (2005). Comparison of the effects of bilateral orbital prefrontal cortex lesions and amygdala lesions on emotional responses in rhesus monkeys. *J. Neurosci.* 25 8534–8542. 10.1523/JNEUROSCI.1232-05.2005 16162935 PMC6725674

[B36] Kalin N. H. Shelton S. E. Davidson R. J. (2004). The role of the central nucleus of the amygdala in mediating fear and anxiety in the primate. *J. Neurosci.* 24 5506–5515. 10.1523/JNEUROSCI.0292-04.2004 15201323 PMC6729317

[B37] Kalin N. H. Shelton S. E. Davidson R. J. Kelley A. E. (2001). The primate amygdala mediates acute fear but not the behavioral and physiological components of anxious temperament. *J. Neurosci.* 21 2067–2074. 10.1523/JNEUROSCI.21-06-02067.2001 11245690 PMC6762619

[B38] Kawai N. Koda H. (2016). Japanese monkeys (*Macaca fuscata*) quickly detect snakes but not spiders: evolutionary origins of fear-relevant animals. *J. Comp. Psychol.* 130 299–303. 10.1037/com0000032 27078076

[B39] Koller K. Rafal R. D. Platt A. Mitchell N. D. (2019). Orienting toward threat: contributions of a subcortical pathway transmitting retinal afferents to the amygdala via the superior colliculus and pulvinar. *Neuropsychologia* 128 78–86. 10.1016/j.neuropsychologia.2018.01.027 29410291

[B40] Kusama T. Mabuchi M. (1970). *Stereotaxic Atlas of the Brain of Macaca fuscata.* Tokyo: Tokyo University Press.

[B41] Langton S. R. Law A. S. Burton A. M. Schweinberger S. R. (2008). Attention capture by faces. *Cognition* 107 330–342. 10.1016/j.cognition.2007.07.012 17767926

[B42] Le Q. V. Isbell L. A. Matsumoto J. Le V. Q. Nishimaru H. Hori E. (2016). Snakes elicit earlier, and monkey faces, later, gamma oscillations in macaque pulvinar neurons. *Sci. Rep.* 6:20595. 10.1038/srep20595 26854087 PMC4744932

[B43] Le Q. V. Isbell L. A. Nguyen M. N. Matsumoto J. Hori E. Maior R. S. (2013). Pulvinar neurons reveal neurobiological evidence of past selection for rapid detection of snakes. *Proc. Natl. Acad. Sci. U. S. A.* 110 19000–19005. 10.1073/pnas.1312648110 24167268 PMC3839741

[B44] Machado C. J. Kazama A. M. Bachevalier J. (2009). Impact of amygdala, orbital frontal, or hippocampal lesions on threat avoidance and emotional reactivity in nonhuman primates. *Emotion* 9 147–163. 10.1037/a0014539 19348528 PMC4410686

[B45] Maior R. S. Hori E. Barros M. Teixeira D. S. Tavares M. C. H. Ono T. (2011). Superior colliculus lesions impair threat responsiveness in infant capuchin monkeys. *Neurosci. Lett.* 504 257–260. 10.1016/j.neulet.2011.09.042 21970966

[B46] Maior R. S. Hori E. Tomaz C. Ono T. Nishijo H. (2010). The monkey pulvinar neurons differentially respond to emotional expressions of human faces. *Behav. Brain Res.* 215 129–135. 10.1016/j.bbr.2010.07.009 20643164

[B47] Martinez R. C. Carvalho-Netto E. F. Ribeiro-Barbosa E. R. Baldo M. V. Canteras N. S. (2011). Amygdalar roles during exposure to a live predator and to a predator-associated context. *Neuroscience* 172 314–328. 10.1016/j.neuroscience.2010.10.033 20955766

[B48] Martínez-Íñigo L. Engelhardt A. Agil M. Pilot M. Majolo B. (2021). Intergroup lethal gang attacks in wild crested macaques, *Macaca nigra*. *Anim. Behav.* 180 81–91. 10.1016/j.anbehav.2021.08.002

[B49] Mason W. A. Capitanio J. P. Machado C. J. Mendoza S. P. Amaral D. G. (2006). Amygdalectomy and responsiveness to novelty in rhesus monkeys (*Macaca mulatta*): generality and individual consistency of effects. *Emotion* 6 73–81. 10.1037/1528-3542.6.1.73 16637751

[B50] Matsuda K. (1996). Measurement system of the eye positions by using oval fitting of a pupil. *Neurosci. Res. Suppl.* 25:270.

[B51] McFadyen J. Dolan R. J. Garrido M. I. (2020). The influence of subcortical shortcuts on disordered sensory and cognitive processing. *Nat. Rev. Neurosci.* 21 264–276. 10.1038/s41583-020-0287-1 32269315

[B52] McFadyen J. Mattingley J. B. Garrido M. I. (2019). An afferent white matter pathway from the pulvinar to the amygdala facilitates fear recognition. *Elife* 8:e40766. 10.7554/eLife.40766 30648533 PMC6335057

[B53] McGarry L. M. Carter A. G. (2017). Prefrontal Cortex Drives Distinct Projection Neurons in the Basolateral Amygdala. *Cell Rep.* 21 1426–1433. 10.1016/j.celrep.2017.10.046 29117549 PMC5714295

[B54] Méndez-Bértolo C. Moratti S. Toledano R. Lopez-Sosa F. Martínez-Alvarez R. Mah Y. H. (2016). A fast pathway for fear in human amygdala. *Nat. Neurosci.* 19 1041–1049. 10.1038/nn.4324 27294508

[B55] Meunier M. Bachevalier J. Murray E. A. Málková L. Mishkin M. (1999). Effects of aspiration versus neurotoxic lesions of the amygdala on emotional responses in monkeys. *Eur. J. Neurosci.* 11 4403–4418. 10.1046/j.1460-9568.1999.00854.x 10594668

[B56] Morris J. S. DeGelder B. Weiskrantz L. Dolan R. J. (2001). Differential extrageniculostriate and amygdala responses to presentation of emotional faces in a cortically blind field. *Brain* 124 1241–1252. 10.1093/brain/124.6.1241 11353739

[B57] Nelson E. E. Shelton S. E. Kalin N. H. (2003). Individual differences in the responses of naïve rhesus monkeys to snakes. *Emotion* 3 3–11. 10.1037/1528-3542.3.1.3 12899313

[B58] Nishijo H. Ono T. Nishino H. (1988a). Single neuron responses in amygdala of alert monkey during complex sensory stimulation with affective significance. *J. Neurosci.* 8 3570–3583. 10.1523/JNEUROSCI.08-10-03570.1988 3193171 PMC6569584

[B59] Nishijo H. Ono T. Nishino H. (1988b). Topographic distribution of modality-specific amygdalar neurons in alert monkey. *J. Neurosci.* 8 3556–3569. 10.1523/JNEUROSCI.08-10-03556.1988 3193170 PMC6569600

[B60] Öhman A. Lundqvist D. Esteves F. (2001). The face in the crowd revisited: a threat advantage with schematic stimuli. *J. Pers. Soc. Psychol.* 80 381–396. 10.1037/0022-3514.80.3.381 11300573

[B61] Öhman A. Mineka S. (2001). Fears, phobias, and preparedness: toward an evolved module of fear and fear learning. *J. Pers. Soc. Psychol.* 80 381–396. 11488376 10.1037/0033-295x.108.3.483

[B62] Öhman A. Soares S. C. Juth P. Lindström B. Esteves F. (2012). Evolutionary derived modulations of attention to two common fear stimuli: serpents and hostile humans. *J. Cogn. Psychol.* 24 17–32.

[B63] Pegna A. J. Khateb A. Lazeyras F. Seghier M. L. (2005). Discriminating emotional faces without primary visual cortices involves the right amygdala. *Nat. Neurosci.* 8 24–25. 10.1038/nn1364 15592466

[B64] Pitcher D. Japee S. Rauth L. Ungerleider L. G. (2017). The Superior Temporal Sulcus Is Causally Connected to the Amygdala: a Combined TBS-fMRI Study. *J. Neurosci.* 37 1156–1161. 10.1523/JNEUROSCI.0114-16.2016 28011742 PMC5296794

[B65] Railo H. Karhu V. M. Mast J. Pesonen H. Koivisto M. (2016). Rapid and accurate processing of multiple objects in briefly presented scenes. *J. Vis.* 16:8. 10.1167/16.3.8 26849070

[B66] Root C. M. Denny C. A. Hen R. Axel R. (2014). The participation of cortical amygdala in innate, odour-driven behaviour. *Nature* 515 269–273. 10.1038/nature13897 25383519 PMC4231015

[B67] Rotshtein P. Vuilleumier P. Winston J. Driver J. Dolan R. (2007). Distinct and convergent visual processing of high and low spatial frequency information in faces. *Cereb. Cortex* 17 2713–2724. 10.1093/cercor/bhl180 17283203 PMC2600423

[B68] Sackett G. P. (1966). Monkeys reared in isolation with pictures as visual input: evidence for an innate releasing mechanism. *Science* 154 1468–1473. 10.1126/science.154.3755.1468 4958618

[B69] Schaefer H. S. Larson C. L. Davidson R. J. Coan J. A. (2014). Brain, body, and cognition: neural, physiological and self-report correlates of phobic and normative fear. *Biol. Psychol.* 98 59–69. 10.1016/j.biopsycho.2013.12.011 24561099 PMC4251669

[B70] Shepard R. N. (1962). The analysis of proximities: multidimensional scaling with an unknown distance function. *Psychometrika* 27, 125–140.

[B71] Simoncelli E. P. Olshausen B. A. (2001). Natural image statistics and neural representation. *Annu. Rev. Neurosci.* 24 1193–1216. 10.1146/annurev.neuro.24.1.1193 11520932

[B72] Soares S. C. Maior R. S. Isbell L. A. Tomaz C. Nishijo H. (2017). Fast detector/first responder: interactions between the superior colliculus-pulvinar pathway and stimuli relevant to primates. *Front. Neurosci.* 11:67. 10.3389/fnins.2017.00067 28261046 PMC5314318

[B73] Sugita Y. (2008). Face perception in monkeys reared with no exposure to faces. *Proc. Natl. Acad. Sci. U. S. A.* 8 394–398. 10.1073/pnas.0706079105 18172214 PMC2224224

[B74] Tamietto M. de Gelder B. (2010). Neural bases of the non-conscious perception of emotional signals. *Nat. Rev. Neurosci.* 11 697–709. 10.1038/nrn2889 20811475

[B75] Tamietto M. Pullens P. de Gelder B. Weiskrantz L. Goebel R. (2012). Subcortical connections to human amygdala and changes following destruction of the visual cortex. *Curr. Biol.* 22 1449–1455. 10.1016/j.cub.2012.06.006 22748315

[B76] Tazumi T. Hori E. Maior R. S. Ono T. Nishijo H. (2010). Neural correlates to seen gaze-direction and head orientation in the macaque monkey amygdala. *Neuroscience* 169 287–301. 10.1016/j.neuroscience.2010.04.028 20412835

[B77] Troiani V. Price E. T. Schultz R. T. (2014). Unseen fearful faces promote amygdala guidance of attention. *Soc. Cogn. Affect. Neurosci.* 9 133–140. 10.1093/scan/nss116 23051897 PMC3907921

[B78] Vuilleumier P. Armony J. L. Driver J. Dolan R. J. (2003). Distinct spatial frequency sensitivities for processing faces and emotional expressions. *Nat. Neurosci.* 6 624–631. 12740580 10.1038/nn1057

[B79] Waleszczyk W. J. Wang C. Benedek G. Burke W. Dreher B. (2004). Motion sensitivity in cat’s superior colliculus: contribution of different visual processing channels to response properties of collicular neurons. *Acta Neurobiol. Exp.* 4 209–228. 10.55782/ane-2004-1507 15366254

[B80] Willenbockel V. Sadr J. Fiset D. Horne G. O. Gosselin F. Tanaka J. W. (2010). Controlling low-level image properties: the SHINE toolbox. *Behav. Res. Methods* 42 671–684. 10.3758/BRM.42.3.671 20805589

[B81] Yoshida M. Itti L. Berg D. J. Ikeda T. Kato R. Takaura K. (2012). Residual attention guidance in blindsight monkeys watching complex natural scenes. *Curr. Biol.* 22 1429–1434. 10.1016/j.cub.2012.05.046 22748317

