# Supplementary material for: Fast Detection of Snakes and Emotional Faces in the Macaque Amygdala
Source: Front Behav Neurosci. 2022 Mar 21;16:839123. doi: 10.3389/fnbeh.2022.839123 (PMC8979552; doi:10.3389/fnbeh.2022.839123)
Supplement: Supplementary file 1 [file Data_Sheet_1.pdf]

Fast detection of snakes and emotional faces in the monkey amygdala

Dinh et al.

Supplementary information

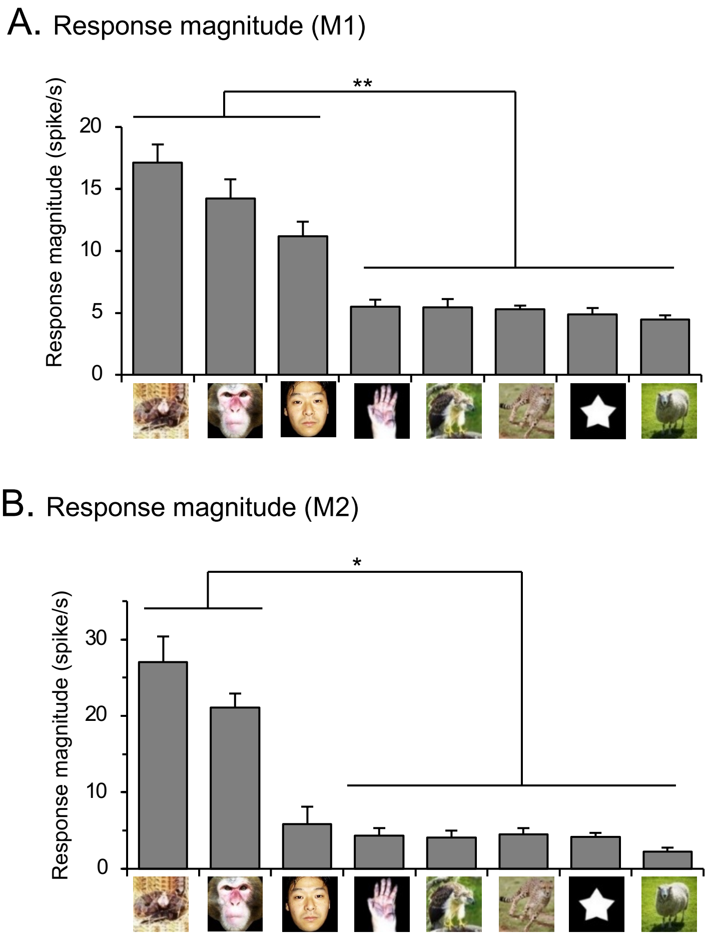

Supplementary Figure 1. Comparison of response magnitudes among the eight categories of the visual stimuli in each monkey.
(A) Comparison of response magnitudes in Monkey1 (M1). There was a significant main effect of stimulus category [repeated measures one-way ANOVA: $F(3.891, 338.5) = 28.87$, $p<0.0001$]. **, $p<0.01$ (Bonferroni test). (B) Comparison of response magnitudes in Monkey2 (M2). There was a significant main effect of stimulus category [repeated measures one-way ANOVA: $F(1.966, 11.79) = 29.30$, $p <0.0001$]. *, $p<0.05$ (Bonferroni test).

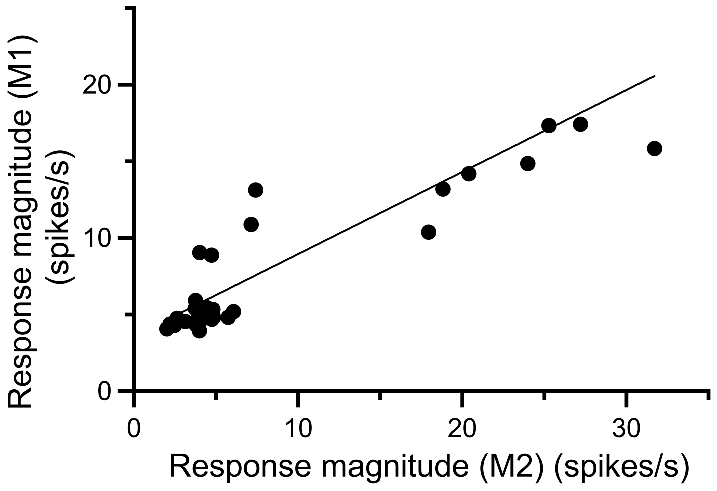

Supplementary Figure 2. Linear correlation of response magnitudes between the Monkey1 (M1) and Monkey2 (M2).

Response magnitudes to the same 32 stimuli in the amygdala of the two monkeys are plotted. There was a significant positive correlation between the two monkeys [$F(1, 30) = 7 1.02$, $p < 0.0001$].

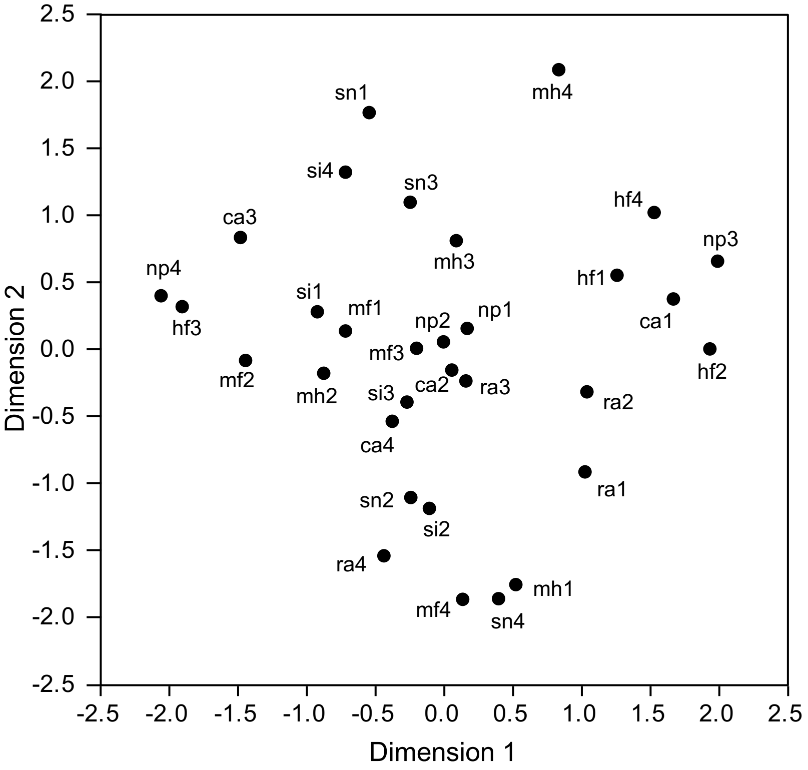

Supplementary Figure 3. Distributions of the 32 visual stimuli in a 2D space resulting from MDS using responses of the 95 amygdalar neurons to these stimuli in epoch 1. No stimulus separation was observed. For abbreviations of the stimuli, see Fig. 1Aa.

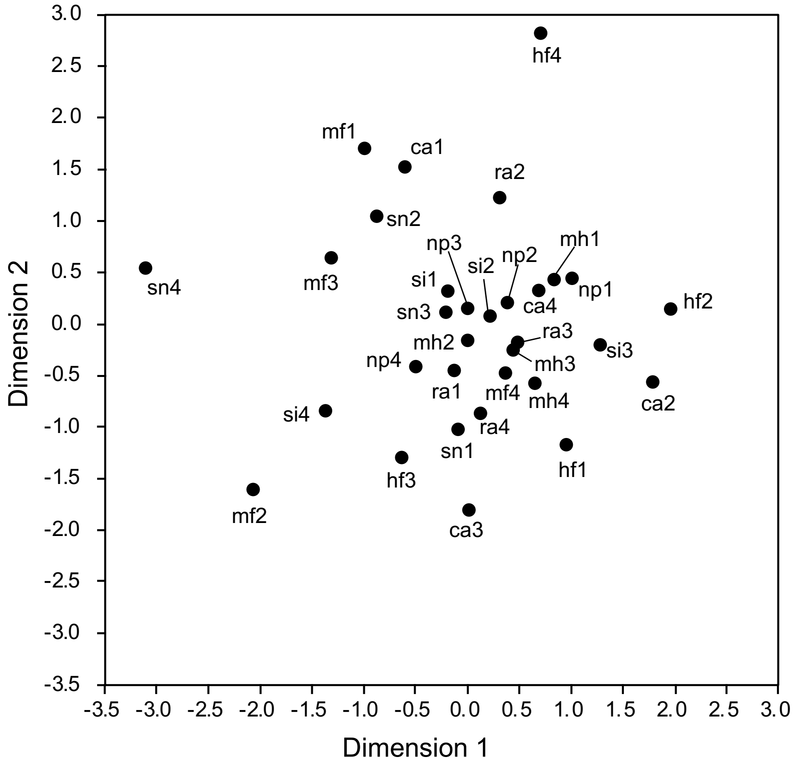

Supplementary Figure 4. Distributions of the 32 visual stimuli in a 2D space resulting from MDS using responses of the 95 amygdalar neurons to these stimuli in epoch 4. No stimulus separation was observed. For abbreviations of the stimuli, see Fig. 1Aa.
