# Supplementary material for: Fast Detection of Snakes and Emotional Faces in the Macaque Amygdala
Source: Front Behav Neurosci. 2022 Mar 21;16:839123. doi: 10.3389/fnbeh.2022.839123 (PMC8979552; doi:10.3389/fnbeh.2022.839123)
Supplement: Supplementary file 2 [file Table_1.pdf]

Fast detection of snakes and emotional faces in the monkey amygdala

Dinh et al.

Supplementary information (tables)

Supplementary Table 1. Results of one-way ANOVAs among the five categories (original snakes, adjusted raptors, adjusted human faces, adjusted monkey faces, and adjusted carnivores).

			ANOVA results	
			F(4,15)-values	p-values
Color histograms	Red	bin: 0.00-0.25	0.236249856	0.913518009
		bin: 0.25-0.50	0.728040545	0.586543705
		bin: 0.50-0.75	0.030198612	0.998035064
		bin: 0.75-1.00	0.011916594	0.999684519
	Green	bin: 0.00-0.25	0.554500726	0.698921369
		bin: 0.25-0.50	0.098319789	0.981379682
		bin: 0.50-0.75	0.002875815	0.999981345
		bin: 0.75-1.00	0.00412696	0.999961663
	Blue	bin: 0.00-0.25	1.426459565	0.273317008
		bin: 0.25-0.50	0.49406085	0.740340734
		bin: 0.50-0.75	0.041578221	0.996345049
		bin: 0.75-1.00	0.124869561	0.971225194
Michelson contrast			1	0.438000811
Power spectrum	Low freq. (1-8)	Red	1.801000244	0.181203957
		Green	1.953911767	0.153640659
		Blue	1.145773908	0.373022713
	High freq. (9-113)	Red	0.433822576	0.78214687
		Green	0.447238394	0.772819971
		Blue	0.397795637	0.807148562

Supplementary Table 2. Results (p-values) of t-tests (original snakes vs. adjusted images).

			Categories of the adjusted images			
			Adjusted raptors	Adjudted human faces	Adjusted monkey faces	Adjuted carnivores
Color histogram	Red	bin: 0.00-0.25	0.662	0.603	0.663	0.663
		bin: 0.25-0.50	0.420	0.415	0.452	0.401
		bin: 0.50-0.75	0.876	0.940	0.916	0.847
		bin: 0.75-1.00	0.994	0.936	0.969	0.967
	Green	bin: 0.00-0.25	0.502	0.450	0.491	0.496
		bin: 0.25-0.50	0.793	0.763	0.742	0.774
		bin: 0.50-0.75	0.996	0.995	0.956	0.976
		bin: 0.75-1.00	0.955	0.943	0.977	0.975
	Blue	bin: 0.00-0.25	0.254	0.239	0.312	0.306
		bin: 0.25-0.50	0.451	0.526	0.582	0.524
		bin: 0.50-0.75	0.830	0.967	0.935	0.872
		bin: 0.75-1.00	0.932	0.771	0.949	0.948
Michelson contrast			0.356	0.356	0.356	0.356
Power spectrum	Low freq. (1-8)	Red	0.230	0.226	0.224	0.232
		Green	0.212	0.207	0.210	0.219
		Blue	0.337	0.316	0.318	0.333
	High freq. (9-113)	Red	0.518	0.600	0.563	0.497
		Green	0.517	0.580	0.544	0.498
		Blue	0.550	0.679	0.571	0.508
